# Accuracy of a surface-based fusion method when integrating digital models and the cone beam computed tomography scans with metal artifacts

**DOI:** 10.1038/s41598-022-11677-9

**Published:** 2022-05-16

**Authors:** Bingshuang Zou, Jung-Hoon Kim, So-Hyun Kim, Tae-Hyun Choi, Yonsoo Shin, Yoon-Ah Kook, Nam-Ki Lee

**Affiliations:** 1 Division of Orthodontics, Faculty of Dentistry, University of British Columbia, Vancouver, BC Canada; 2 Department of Orthodontics, National Health Insurance Service Ilsan Hospital, Goyang, South Korea; 3 Department of Orthodontics, Seoul National University Bundang Hospital, Seongnam, South Korea; 4 Department of Orthodontics, Seoul St. Mary’s Hospital, College of Medicine, The Catholic University of Korea, Seoul, South Korea

**Keywords:** Outcomes research, Dentistry, Three-dimensional imaging

## Abstract

The aim of this study was to evaluate the intra- and inter-observer reliability of maxillary digital dental model integration into cone-beam computed tomography (CBCT) scans to reconstruct three-dimensional (3D) skeletodental models for orthognathic patients. This retrospective study consisted of CBCT and digital maxillary dentition images of 20 Class III orthognathic patients. After two repeated fusions of digital cast images with reconstructed CBCT images by a digital engineer and an orthodontist respectively, the 3D coordinate values of the canines, first molars, and central incisors were evaluated. The intra- and inter-observer reliability of 3D positions of maxillary teeth were compared using intraclass correlation coefficients (ICCs). Intra-observer reliability of x-, y-, and z-coordinate values of maxillary teeth showed significant and excellent agreement in an engineer (0.946 ≤ ICC ≤ 1.000) and an orthodontist (0.876 ≤ ICC ≤ 1.000). The inter-observer reliability of the y- and z-coordinates of each tooth was significantly excellent or good, but that of the x-coordinates showed insignificantly poor to moderate agreement. This study showed that the integration of maxillary digital models into CBCT scans was clinically reliable. However, considering the low inter-observer reliability on the x-coordinates of dentition, clinical experience and repeated learning are needed for accurate application of digital skeletodental model in orthognathic patients.

## Introduction

Cone-beam computed tomography (CBCT) has been applied for orthodontic diagnosis and the treatment planning of three-dimensional (3D) craniofacial skeletal abnormalities, impacted teeth, and virtual simulation of orthognathic surgery^1,2^. CBCT with the development of related software can provide clinicians with more useful information on the 3D positions of anatomical features.

However, many studies have reported that the representation of dentition on CBCT is not reliable^3–5^. Digital study models generated from CBCT had less accuracy for all dental measurements compared with those acquired from plaster models^6^. Therefore, the replacement of the dentition from CBCT scans with digital dental models is more likely to be needed^7–10^.

In addition, digital models, which are taken by extra- or intra-oral scanners, are known to be reliable and present as accurate an interocclusal relationship as traditional plaster models, with high accuracy and reproducibility^11–13^. Some studies reported that orthodontic measurements from digital models were comparable to those from plaster models^13–16^.

With the increased accuracy of digital dental images, the configuration (positioning) of these images on reconstructed facial CBCT images is important for accurate virtual simulation and planning for orthognathic surgery, actual surgical splint fabrication, and accurate surgery results^17–19^. Previous studies have introduced the accuracy of various methods using fiducial markers or triple CBCT scans, which replaced the dentition area of CBCT scans with digitally scanned dental images^10,20–23^. Most of them reported less than 0.3 mm of registration errors and high accuracy for the replacement of dentition with CBCT, despite their different superimposition methods.

However, to the best of our knowledge, there have been few studies about intra- and inter-rater reliability and reproducibility of the implementation of digital dentition into reconstructed CBCT image in patients undergoing orthognathic surgery.

Therefore, it is necessary to evaluate the reliability of merging digital cast images with facial CBCT scans. The null hypothesis is that there would be no difference in the reproducibility of maxillary dentition position when integrating digital dental arches into CBCT scans. The aims of this study were to evaluate the intra-observer and inter-observer reliability of integration of maxillary digital dental models into facial CBCT scans to reconstruct 3D skeletodental models in orthognathic patients and to compare the positional deviations of teeth after repeated registrations between a digital engineer and an orthodontist.

## Results

### Intra- and inter-observer reliability of x-, y-, and z-coordinates of each tooth during merging of maxillary dentition scanned images and facial CBCT by a digital engineer and an orthodontist

As shown in Table 1, the intra-observer reliability of x-, y-, and z-coordinate values of maxillary teeth was significant and almost perfect for an engineer (0.946 ≤ ICC ≤ 1.000) and an orthodontist (0.876 ≤ ICC ≤ 1.000), respectively.

**Table 1:** Reliability of x, y, and z coordinates of each tooth during immerging of digital maxillary cast images and facial CBCTs.

Coordinates	Intra-observer reliability analysis								Inter-observer reliability analysis between an engineer and an orthodontist			
	An engineer				An orthodontist							
	ICC	95% CI		*P*	ICC	95% CI		*P*	ICC	95% CI		*P*
		Lower bound	Upper bound			Lower bound	Upper bound			Lower bound	Upper bound	
13x	0.998	0.996	0.999	0.000	0.986	0.964	0.994	0.000	0.438	− 0.425	0.778	0.112
13y	1.000	1.000	1.000	0.000	1.000	0.999	1.000	0.000	0.781	0.449	0.913	0.001
13z	1.000	1.000	1.000	0.000	1.000	1.000	1.000	0.000	0.967	0.917	0.987	0.000
16x	0.999	0.998	1.000	0.000	0.938	0.842	0.975	0.000	0.526	− 0.201	0.813	0.058
16y	1.000	1.000	1.000	0.000	0.999	0.997	1.000	0.000	0.769	0.422	0.908	0.001
16z	1.000	1.000	1.000	0.000	0.996	0.989	0.998	0.000	0.971	0.927	0.988	0.000
23x	1.000	1.000	1.000	0.000	0.991	0.977	0.996	0.000	− 0.056	− 1.560	0.575	0.548
23y	1.000	1.000	1.000	0.000	1.000	1.000	1.000	0.000	0.837	0.593	0.935	0.000
23z	1.000	1.000	1.000	0.000	1.000	0.999	1.000	0.000	0.972	0.929	0.989	0.000
26x	0.999	0.998	1.000	0.000	0.980	0.951	0.992	0.000	0.259	− 0.807	0.703	0.256
26y	1.000	1.000	1.000	0.000	1.000	0.999	1.000	0.000	0.843	0.612	0.937	0.000
26z	1.000	1.000	1.000	0.000	1.000	0.999	1.000	0.000	0.974	0.933	0.990	0.000
U1x	0.946	0.864	0.979	0.000	0.876	0.688	0.951	0.000	0.180	− 0.802	0.655	0.318
U1y	1.000	1.000	1.000	0.000	1.000	0.999	1.000	0.000	0.803	0.494	0.922	0.001
U1z	1.000	1.000	1.000	0.000	1.000	1.000	1.000	0.000	0.978	0.946	0.991	0.000

*ICC* intraclass correlation coefficients, *13/23* the cusp of maxillary right/left canine, *16/26* the mesio-buccal cusp of maxillary right/left first molar, *U1* the contact point between the maxillary central incisors, *CI* confidence interval; ICC > 0.8/0.6/0.4/0.2 or ≤ 0.2 represent almost perfect, substantial, moderate, mediocre, or low strength of agreement, respectively.

**Figure 1:**
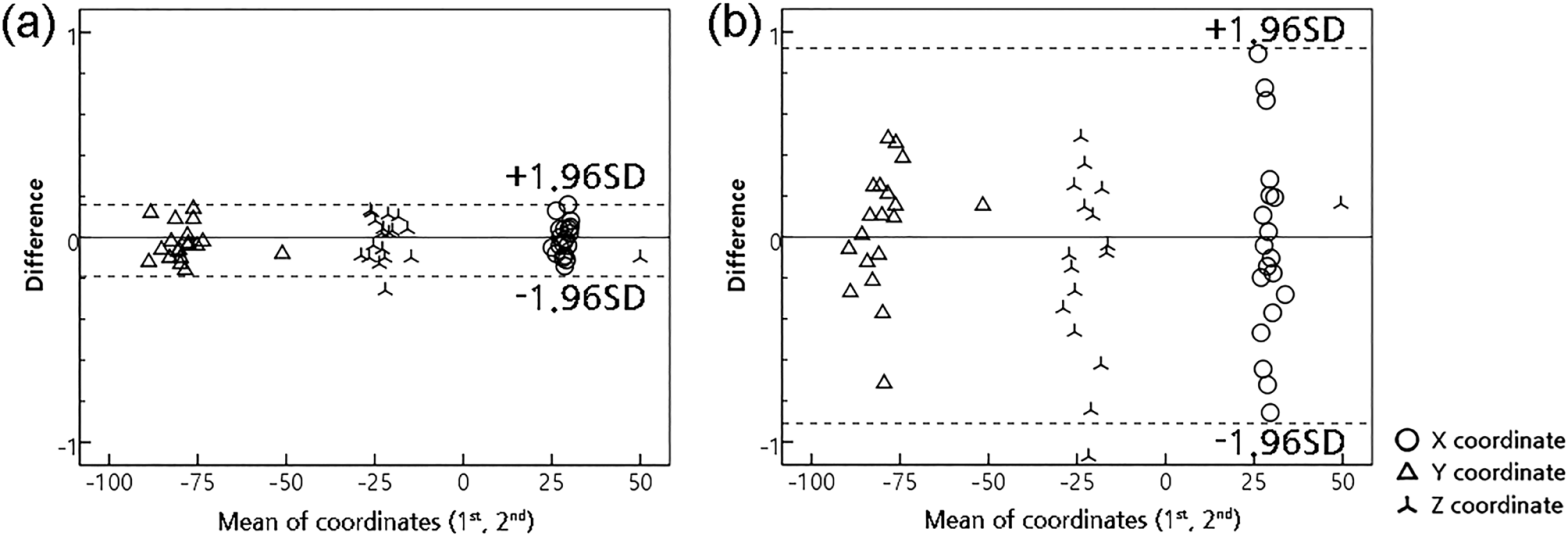
An example of Bland–Altman plot of the x, y, z coordinates of #26 between the first and second registration of maxillary digital cast image and facial CBCT. (**a**) Digital engineer, (**b**) Orthodontist.

The inter-observer reliability of the y- and z-coordinate values of maxillary teeth was significant and presented almost perfect or substantial, while that of x-coordinate values of maxillary teeth was nonsignificant and showed poor to moderate agreement. In addition, Bland–Altman plots showed a larger registration error of teeth on the X-axis compared to those of the y- and z-axes between a digital engineer and an orthodontist (Fig. 1).

### Reproducibility of registration of x-, y-, and z-coordinates of each tooth of maxillary dentition scanned image and facial CBCT by a digital engineer and an orthodontist

As shown in Table 2, the mean differences from the repeated measurements of each tooth on the x-axis ranged from − 0.020 to 0.006 mm for an engineer and from − 0.210 to 0.013 mm for an orthodontist. The mean differences ranged from − 0.036 to 0.027 mm on the y-axis for an engineer and from − 0.045 to 0.081 mm for an orthodontist and ranged from − 0.015 to 0.005 mm on the z-axis for an engineer and from − 0.564 to 0.136 mm for an orthodontist. However, there was no significant difference for each tooth position of the first and second registrations for each observer or between the engineer and orthodontist.

**Table 2:** Descriptive statistics showing the mean differences in x, y, and z coordinates of each tooth between first registration and second registration of digital maxillary cast images and facial CBCTs in a digital engineer and an orthodontist.

Coordinates	△ (1st–2nd) in a digital engineer [unit, mm]			△ (1st–2nd) in an orthodontist [unit, mm]			*P*^†^
	Mean	S.D	P*	Mean	S.D	P*	
13x	− 0.007	0.090	0.980	− 0.104	0.330	0.926	0.221
13y	0.023	0.078	0.982	− 0.045	0.293	0.934	0.324
13z	0.005	0.100	0.978	0.043	0.455	0.898	0.713
16x	− 0.015	0.086	0.981	− 0.210	0.985	0.780^a^	0.390
16y	0.027	0.074	0.983	− 0.005	0.535	0.880	0.794
16z	0.005	0.102	0.977	− 0.564	2.136	0.522^a^	0.255
23x	− 0.020	0.086	0.981	− 0.079	0.280	0.937	0.406
23y	− 0.012	0.101	0.977	0.002	0.237	0.947	0.825
23z	− 0.010	0.096	0.978	0.136	0.470	0.895	0.181
26x	− 0.011	0.081	0.982	− 0.068	0.474	0.894	0.577
26y	− 0.036	0.087	0.981	0.025	0.301	0.933	0.395
26z	− 0.015	0.102	0.977	0.038	0.592	0.868	0.692
U1x	0.006	0.105	0.976	0.013	0.256	0.943	0.895
U1y	0.010	0.105	0.976	0.081	0.328	0.927^a^	0.366
U1z	0.002	0.091	0.980	0.070	0.388	0.913	0.460

△ (1st–2nd), the mean difference in x, y, and z coordinates of each tooth between first registration (1st) and second registration (2nd) of digital maxillary cast images and facial CBCT images.

*S.D.* standard deviation.

*Paired *t* test.

^†^Independent *t* test.

^a^Wilcoxon Signed-rank test.

## Discussion

For the virtual planning and clinical application of orthognathic surgery, accurate representation of dentition as well as the maxilla-mandibular complex from CBCT scans is necessary. Most CBCT scans have streak artifacts in the dental portion due to metallic restorations or orthodontic brackets in patients treated with orthognathic surgery^7^. Therefore, the dentition part from CBCT needs to be replaced by a digital dental model^7–10^.

This study evaluated the intra- and inter-observer reliability of the integrated maxillary digital model images and CBCT scans to reconstruct 3D skeletodental models in skeletal class III patients treated with orthognathic surgery.

To date, previous studies have introduced various methods including fiducial markers or triple CBCT scans, which replaced the dentition areas of CBCT scans with digitally scanned dental images^10,20–23^. In contrast, in our study, the surface-based fusion method (best-fit method) with the iterative closest point algorithm was used to superimpose a digital cast scan and CBCT scan in each sample^4,24–26^.

The intra-observer reliability of x-, y-, and z-axis positions of maxillary dentition from the repeated fusion of digitally scanned dental images with CBCT scans showed significant and excellent agreement for an engineer and an orthodontist (Table 1). This means that the measurement points, which were the contact points of the central incisors and the cusp tips of the canines and first molars, were defined consistently by each observer. Kim et al. reported that the reliable identification areas of 3D landmarks were distinct structures like cusp tips and points positioned in the midline^27^.

**Figure 2:**
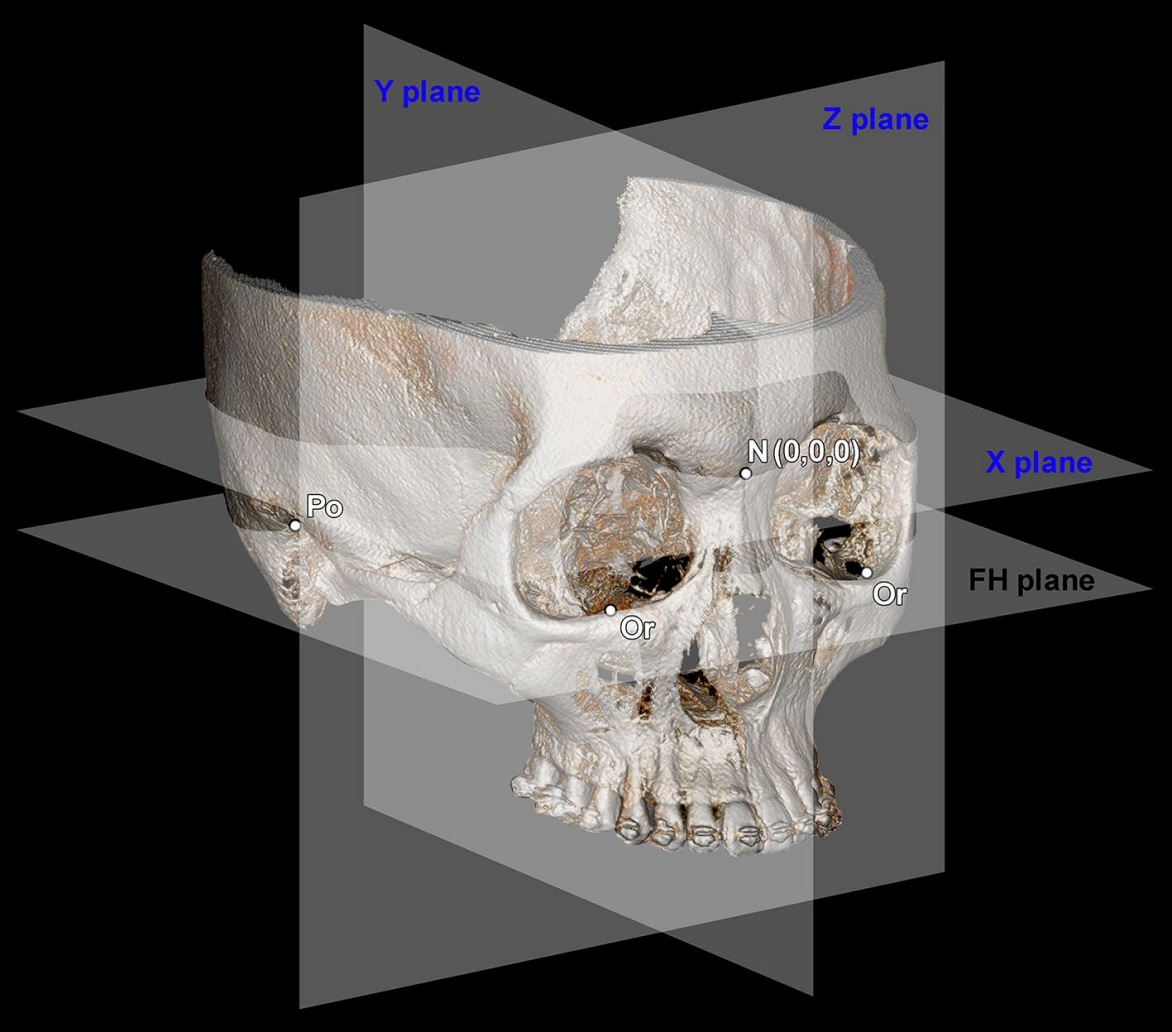
The DICOM data from CBCT were converted to stereolithography format, oriented, and reconstructed following reference planes. The X plane is the plane passing the nasion (N), which is parallel to the Frankfort horizontal (FH) plane passing through the left and right orbitales (Or) and the right porion (Po). The Y plane is the plane passing through the N and basion, while perpendicular to the X plane, and the Z plane is perpendicular to the X- and Y-planes, setting the plane through the N (0, 0, 0).

However, inter-observer reliability proved to be different in two groups. Although the reliability of each tooth on the y- and z-axes showed significantly excellent or good agreement, those on the x-axis showed insignificantly varied agreement levels such as poor, fair, and moderate. This means that low reliability between the two examiners could occur in the tooth positions on the x-axis, which represents the right and left directions, compared to the reliability on y- and z-axes (Table 1, Fig. 2). This error might be associated with streak artifacts in the dentition area of the CBCT images. These artifacts, which are due to orthodontic brackets mainly located on the buccal side of anterior and posterior teeth and partial attachments on the palatal side in orthognathic samples of this study, could cause difficulty during the integration by limiting the surface as a reference for registration. Specifically, numerous buccal and palatal artifacts in the molar area seem to cause a registration error on the x-axis. Therefore, for clinical application in the orthognathic patient, clinicians should be aware that operator errors on the horizontal plane can occur.

With regard to the replacement of the dentition areas of CBCT scans with digital dental images, Uechi et al. reported root-mean-square errors of 0.1 to 0.5 mm, when registered with ceramic balls^20^. Swennen et al. showed mean errors ranging from 0.1 to 0.3 mm, when using gutta-percha cones^21–23^.

On the other hand, in our study, the surface-based fusion method (best-fit method) was used with the iterative closest point algorithm^4,25,28^. Noh et al. suggested that registration with the buccal and lingual tooth surfaces should be used as much as possible to increase the accuracy of the integration of laser-scanned dental images into the maxillofacial CBCT scans^4^. They presented mean distance errors ranging from 0.27 to 0.33 mm between the surface points on the two images. In addition, Lin et al. reported that the mean errors of dentition obtained digitally in 14 patients ranged from 0.10 to 0.43 mm^12^. Rangel et al. reported a mean absolute distance of 0.39 mm in the upper jaw, when comparing two methods to integrate digital dental models into CBCT scans^5^. In this study, each tooth after repeated registrations showed mean deviations that ranged from − 0.04 to 0.03 mm for an engineer and from − 0.56 to 0.14 mm for an orthodontist (Table 2). This deviation by an orthodontist was larger than that of a digital engineer, but there was no significant difference between the two groups. This suggests that it may take repeated experiences and a learning curve to reduce 3D positioning errors and to increase the reproducibility of integrating digital dental images into CBCT scan images.

To date, most studies using surface-based fusion evaluated the registration accuracy in adults with normal occlusion and no crown restorations, or with Class II or III malocclusion with no brackets^4,12^. Almutairi et al. measured the errors related to the fusion of intraoral scan images with CBCT using six dried skulls with orthodontic brackets^9^. In contrast, our study evaluated the reproducibility of each tooth position after the fusion of digital dental images with CBCT scans in actual surgery patients with orthodontic brackets. Although Kim et al. evaluated the accuracy of the maxillary repositioning after orthognathic surgery using computer-aided and surgical simulation, they did not report on the reproducibility of reference points of the teeth before surgery^17^.

This retrospective study had some limitations, such as a small sample size and no registration of mandibular dentition. Further studies are needed to evaluate the differences between the positions of maxillary and mandibular teeth registered on facial CBCT scans for digital virtual surgery and their positions mounted for conventional model surgery. Also, the accuracy of results after actual surgery could be evaluated compared to the simulation of virtual surgery.

## Conclusion

We failed to reject the null hypothesis that there would be no difference in the reproducibility of maxillary dentition position when integrating digital dental arches into CBCT scans.

This study showed that the integration of maxillary digital models into CBCT scans was clinically reliable. However, considering the low to moderate inter-observer reliability on the x-coordinates of dentition, it is necessary for clinicians to have clinical experience and repeated learning for accurate application of digital skeletodental model in orthognathic patients.

## Materials and methods

This retrospective study was reviewed and approved by the Institutional Review Board of Seoul National University Bundang Hospital (B-1911/576-105) and the Institutional Review Board of the University of British Columbia (H19-03765). A total of 20 skeletal Class III adult patients (7 males and 13 females; age, 21.7 ± 4.0 years) were included, who were treated with either one-jaw or double-jaw orthognathic surgery at Seoul National University Bundang Hospital from March 2016 to October 2019. All patients were selected according to the following inclusion criteria: (1) ANB < 0°; (2) had a full set of pre-surgical record, including digital casts and CBCT scans, to fabricate the surgical splint; (3) had fixed edgewise appliances in place when the records were taken. Exclusion criteria were: (1) CBCT scans did not include the whole craniofacial area; (2) had clear aligner therapy.

### Image merging procedure and measurement

All patients underwent CBCT (KODAK 9500, Carestream Health Inc., Rochester, NY, USA), which was obtained with a field of view of 200 mm × 180 mm, a voxel size of 0.2 mm, and exposure conditions of 80 kVp, 15 mA, and 10.8 s. During the CBCT scans, the patients were asked to maintain an upright position. Their heads were positioned in which the Frankfort horizontal planes were parallel to the floor, and stabilized by the ear rods. They were instructed to maintain their teeth in maximum intercuspation. All CBCT scans were saved as Digital Imaging and Communications in Medicine (DICOM) data files. The DICOM data were converted to stereolithography format, oriented, and reconstructed using Geomagic software (Geomagic Qualify 2013^®^, 3D Systems, Morrisville, NC, USA) while following reference planes. The horizontal plane (axial plane; X plane) is the plane passing the nasion, which is parallel to the Frankfort horizontal (FH) plane passing through the left and right orbitales and the right porion. The midsagittal plane (Y plane) is the plane passing through the nasion and basion, while perpendicular to the X plane. The coronal plane (Z plane) is perpendicular to the horizontal and midsagittal planes, setting the plane through the nasion (zero point; 0, 0, and 0) (Fig. 2).

In addition, simultaneously with the acquisition of CBCT for each patient, a conventional impression was taken with alginate (Aroma fine plus normal set, GC Corporation, Tokyo, Japan) to fabricate maxillary and mandibular dental stone casts. For producing digital models, the surface images of the maxillary, mandibular casts and their maximum intercuspation were digitized into standard tessellation language (STL) format using a desktop model scanner (MD-ID0300, Medit Co, Seoul, Korea).

**Figure 3:**
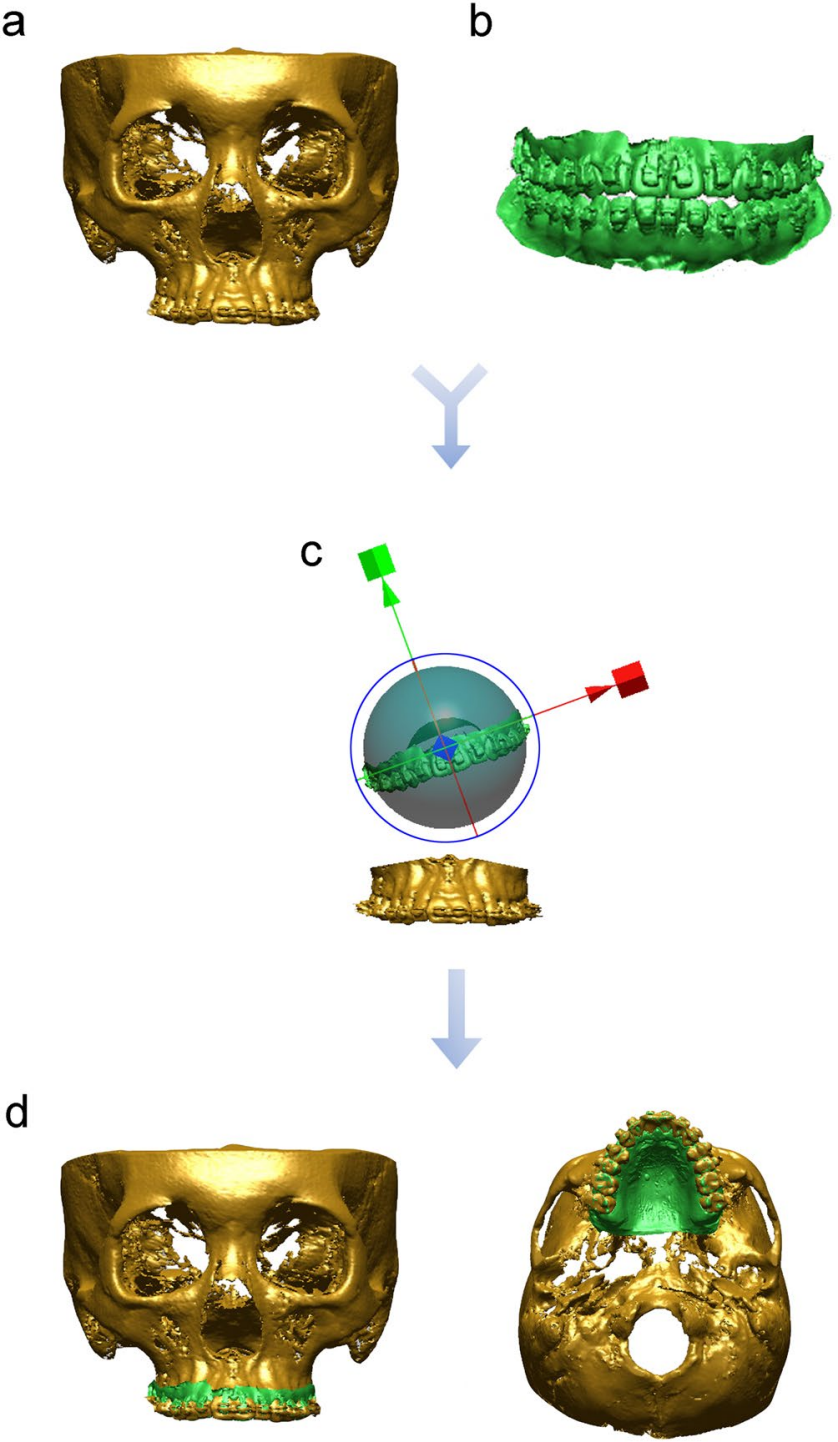
Study workflow of the integration of maxillary digital models into CBCT scans. (**a**) Reconstructed CBCT image, (**b**) digital cast image, (**c**) integration process of maxillary digital cast images into the dental portions of reconstructed CBCT images, (**d**) integrated skeletodental models.

Digital cast images of whole maxillary dentition were merged with the dental portions of reconstructed CBCT images using Geomagic software. First, point-based registration was performed using the cusp tips of the canines and the mesiobuccal cusps of the maxillary first molars in both images. Then, for more precise integration, surface-based registration was performed (Fig. 3). The cusp tips or occluso-buccal surfaces of the teeth above the bracket and the lingual surfaces of the teeth as the registration area were used with the best-fit algorithm^26,28^. This procedure was carried out twice at a two-week interval by 2 examiners (one digital engineer and one orthodontist [J.-H.K.]).

The 3D coordinate values (x, y, and z) of the cusps of the canines, the mesio-buccal cusps of the first molars, and the contact points between the maxillary central incisors were measured in the coordinate system, which is constructed by X-, Y-, and Z-plane through the nasion (zero point; 0, 0, and 0). The differences in the x-, y-, or z-coordinates of each tooth between two repeated fusions, measured by the 2 examiners, were evaluated^13^. In addition, intraclass correlation coefficients (ICCs) were calculated to determine the intra- and inter-examiner reliability of the measurements of 3D positions of maxillary dentition after merging by a digital engineer and an orthodontist, and between the 2 examiners.

### Statistical analysis

Power analysis with correlation ρ H1 = 0.77, α = 0.05, and power (1 − β) = 0.80 showed a sample size requirement of 10 (G*Power v. 3.1.9.7; Heinrich Heine Universität, Dϋsseldorf, Germany)^29^.

All statistical data were analyzed with SPSS software (Version 22.0, IBM, Armonk, NY, USA). Paired t-tests, Wilcoxon’s signed-rank tests, ICC tests and Bland–Altman analyses were performed to evaluate the differences and reproducibility between 3D positions (3D coordinates) of maxillary dentition taken two times by each of the two examiners. The intra-examiner’s reliability and inter-examiner’s reliability were evaluated using ICC as follows: ICC > 0.8/0.6/0.4/0.2 or ≤ 0.2 represent almost perfect, substantial, moderate, mediocre, or low strength of agreement, respectively^30^. Also, the level of significance was set as *P* < 0.05.

### Ethical approval

All procedures performed in studies involving human participants were reviewed and approved by the institutional review board and in accordance with the 1964 Helsinki Declaration and its later amendments or comparable ethical standards.

### Informed consent

For this type of retrospective, non-interventional clinical study, the informed consent was waived by the institutional review board of Seoul National University Bundang Hospital and the institutional review board of the University of British Columbia.
